# Understanding MNPs Behaviour in Response to AMF in Biological Milieus and the Effects at the Cellular Level: Implications for a Rational Design That Drives Magnetic Hyperthermia Therapy toward Clinical Implementation

**DOI:** 10.3390/cancers13184583

**Published:** 2021-09-12

**Authors:** David Egea-Benavente, Jesús G. Ovejero, María del Puerto Morales, Domingo F. Barber

**Affiliations:** 1 Department of Immunology and Oncology, and NanoBiomedicine Initiative, Centro Nacional de Biotecnologia (CNB)-CSIC, Darwin 3, 28049 Madrid, Spain; degea@cnb.csic.es; 2 Department of Energy, Environment and Health, Instituto de Ciencia de Materiales de Madrid (ICMM)-CSIC, Sor Juana Inés de la Cruz 3, 328049 Madrid, Spain; jesus.g.ovejero@csic.es (J.G.O.); puerto@icmm.csic.es (M.d.P.M.); 3 Department of Dosimetry and Radioprotection, Hospital General Universitario Gregorio Marañón, Doctor Esquerdo 46, 28007 Madrid, Spain

**Keywords:** hyperthermia, magnetic hyperthermia, magnetic nanoparticles, magnetic nanoparticle-induced biological effects, clinical trial, new therapies

## Abstract

**Simple Summary:**

Magnetic hyperthermia therapy is an alternative treatment for cancer that complements traditional therapies and that has shown great promise in recent years. In this review, we assess the current applications of this therapy in order to understand why its translation from the laboratory to the clinic has been less smooth than was anticipated, identifying the possible bottlenecks and proposing solutions to the problems encountered.

**Abstract:**

Hyperthermia has emerged as a promising alternative to conventional cancer therapies and in fact, traditional hyperthermia is now commonly used in combination with chemotherapy or surgery during cancer treatment. Nevertheless, non-specific application of hyperthermia generates various undesirable side-effects, such that nano-magnetic hyperthermia has arisen a possible solution to this problem. This technique to induce hyperthermia is based on the intrinsic capacity of magnetic nanoparticles to accumulate in a given target area and to respond to alternating magnetic fields (AMFs) by releasing heat, based on different principles of physics. Unfortunately, the clinical implementation of nano-magnetic hyperthermia has not been fluid and few clinical trials have been carried out. In this review, we want to demonstrate the need for more systematic and basic research in this area, as many of the sub-cellular and molecular mechanisms associated with this approach remain unclear. As such, we shall consider here the biological effects that occur and why this theoretically well-designed nano-system fails in physiological conditions. Moreover, we will offer some guidelines that may help establish successful strategies through the rational design of magnetic nanoparticles for magnetic hyperthermia.

## 1. Introduction: From Cancer to Magnetic Hyperthermia Therapy via Nanomedicine

### 1.1. Cancer

Cancer is a multifactorial disease in which a variety of parameters influence its development, progression or outcome, such as the type of cancer, tissue localization, genetic predisposition, immune status of the patient, etc. For this reason, it is one of the most challenging diseases to treat and develop new and effective therapies, which in turn requires the cooperation of multidisciplinary teams. Many types of therapies have been approved to treat cancer and the specific therapy or a combination of these that patients receive depends on factors like the type or stage of development of the cancer. Traditionally, the most common treatments for cancer involve surgery, radiotherapy and chemotherapy. However, more recently, strategies like immunotherapy have been developed and implemented in combination with these established approaches due to their capacity to improve these treatments. Complementary therapies like stem cell transplant also help restore blood-forming stem cells in patients after particularly harsh treatments. In addition, biomolecular advances have helped us better understand the causes of certain types of cancer, guiding the use of more specific and precise treatments, for example, using biomarkers or genetic studies [1].

### 1.2. Hyperthermia

Hyperthermia (HT) is a cancer treatment strategy first shown to produce benefits in the 1940s when it was contemplated that rising the temperature of a tissue might combat fibrosis and cancer [2]. To treat cancer, HT involves exposing malignant tissues to supraphysiological temperatures [3]. Damage to tumour cells or their death are the main desirable effects of such heating but also, HT may improve tumour antigen presentation, the activation of dendritic and NK cells, and leukocyte trafficking through the endothelium [4]. These are phenomena that enhance the anti-tumour immune response, and that make cancer cells more sensitive to the effects of radiotherapy and chemotherapy. HT is generally considered to be defined as a rise in temperature of the tumour region to between 39 and 43 °C (known also as mild hyperthermia) [3,5], although an increase up to 45 °C may also be considered [6]. However, when the temperature rises above 45 °C the situation is usually referred to as thermal ablation, which may have dramatic side-effects due to the damage caused to normal tissue and the death of healthy cells. For this reason, careful temperature control is necessary during HT treatment. Usually, MHT is administered such a multivalent oncological strategy in combination with other anti-cancer approaches, specifically when HT has been demonstrated to produce an improved synergic effect [7].

HT therapies are mainly classified according to the area of the body treated, which is usually closely related with the method used to increase the temperature (energy source) [6,7].

#### 1.2.1. Whole-Body Hyperthermia

Whole-body HT is the systemic heating of the body in an attempt to obtain benefits treating widely disseminated metastatic cancer. The recommended upper limits are 42 °C maintained for 1 h, or 40 °C in combination with cytotoxic drugs. Technically, this method has important drawbacks, one of which is the need to sedate or submit the patient to general anaesthesia. Moreover, rising the body temperature from 37 °C to 42 °C is a lengthy process (90–180 min). In many cases the side effects of whole-body HT are unacceptable and much effort has been made to develop equipment that can resolve these disadvantages, e.g., IRATHERM-2000, currently in phase I/II clinical trials [8]. However, the success of this approach is limited by the poor balance between risk and benefit, and by the increasing interest in applying the fundamentals of HT in a safer and more specific manner [9].

#### 1.2.2. Regional Hyperthermia

Regional HT is the application of the HT to a whole organ, limb or region but not to a specific tumour area. The most commonly used strategy to raise the temperature in selected regions is perfusion HT. Perfusion HT consists of inducing a heated fluid flow, normally of the patient’s own blood, through a specific area, and it is usually directed at tumours localized to a limb. Nevertheless, there are variants where stomach cancer or other tumours in the abdominal cavity are treated in this way, such as continuous hyperthermia peritoneal perfusion (CHPP). This approach is not technically difficult and it is safe to employ by heating the perfusion fluid up to 43 °C for 2 h. However, this technique alone does not produce outstanding results and only in synergism with cytotoxic drugs are the desired results produced. This combined therapy needs a precise adjustment of certain parameters, such as the flow rate (30–40 mL/min), the cells, pH and O_2_ of the blood perfused, or the amount of drug administered (which will be higher than in more specific therapies) in order to avoid undesirable toxicity [6,10]

#### 1.2.3. Local Hyperthermia

Local HT is based on the controlled heating of a specific tumour zone in an attempt to avoid side-effects in the healthy surrounding area. This approach is based on the application of electromagnetic waves or ultrasound through a physical stimulator, enabling local heat to be applied in an external or an invasive way. The external application operates through micro- or radio-waves directed at a superficial or slightly deeper solid tumour (only a few centimetres below the skin) by an external device (e.g., microwave antennas, radiofrequency electrodes, laser fibres, electromagnetic coils or ultrasound transducers). Alternatively, invasive local HT (or interstitial HT) requires a thin needle or probe to be inserted into the tumour and serve as in situ energy applier. In this case, invasive local HT can be used in a deep tumour but it is restricted to small tumours (less than 5 cm in diameter) located in an accessible organ or tissue, such as the head, neck, bladder or prostate. In general terms, the success of local HT is determined by the tissue characteristics and blood flow, factors on which the energy and heat distribution is strongly dependent. Indeed, the heat distribution is often not as homogenous as would be desired and to resolve this problem, segmented radio-frequency electrodes that allow a three-dimensional control of heating have been evaluated for clinical implementation [6,7,10].

#### 1.2.4. The Drawbacks of Conventional Hyperthermia

Without any doubt, HT is a very promising approach for cancer treatment and various clinical trials support its application for a wide range of different cancers: head, neck, melanoma, sarcoma, breast, glioblastoma multiforme (GBM), bladder, cervix, rectum, oesophagus, lung, mesothelioma and paediatric germ cell tumours [6]. However, since its first clinical application in the 1980s, its implementation has not been as widespread as might have been expected, probably reflecting several of the problems that traditional HT presents.

Firstly, HT requires specific equipment and technically, it is more complex than other more standard therapies like chemotherapy. Currently, there is considerable effort being directed at developing new, improved equipment. Secondly, the limited effectiveness of HT means it cannot compete with the standard protocols of cancer therapy. For an acceptable result, HT must be applied in combination with other therapies, mainly chemotherapy and radiotherapy, producing notable synergic effects. Consequently, HT is often considered as a sensitizing adjuvant therapy more than a cancer therapy by itself [11]. Traditionally, this reduced effectiveness can be explained by the uncontrolled dispersion of the heat, which is in turn caused by the lack of powerful devices to control and monitor the local temperature. In addition, this situation is exacerbated by the physical and physiological handicaps, such as the non-homogeneity of the tissues, the physical distance between the tumour cells to be treated and the heat source, or the thermal dissipation produced by the circulatory system. Thirdly, HT also produces side-effects and the rise in temperature may provoke non-desirable toxicity in healthy tissues or cells, even after local application. This is again due to the heat source not being exactly adjacent to the cancer cells, producing local side-effects. Finally, more studies into thermal biology and how HT affects individual cells molecularly are needed to better understand the process. Above all, it is necessary to assess the thermotolerance that has often been proposed [5,7,10] and to analyse the sensitivity of different types of cancer to temperature.

A general roadmap to address the aforementioned limitations involves improving the real-time temperature control of the tumour region and effectively localizing the induction of heat using a contactless stimulus. Regarding the former, technical developments have been quite successful, for example with Magnetic Resonance Thermometry (MRT). MRT is a MRI (magnetic resonance imaging) based technique that involves non-invasive 3D measurement of temperature distributions that could substitute the currently used invasive thermal probes. Since combining thermometry with MRI was first proposed four decades ago [12], MRT techniques have improved in accuracy and robustness for in vivo applications [13], now reaching pre-clinical stages of development [13,14]. Moreover, new approaches based on nanomedicine have been explored to obtain non-invasive heat sources that can be precisely targeted to the tumour cell in order to avoid side-effects and enhance the effectiveness of HT. Nevertheless, it would be desirable to have a single equipment where in vivo location and MHT could be performed simultaneously. However, limitations intrinsic to the technique (as different magnetic field requirements between MRI and MHT) do not allow a real-time guidance [15].

### 1.3. Nanomedicine: A Trip through the Hyperthermia Based Nanotherapies to Treat Cancer

Medicine is evolving towards more specific and personalised therapies, and HT must also move in this direction. Faced by this challenge, nanomedicine emerges as a very promising alternative to convert HT into a well-implanted and important cancer treatment. In recent decades, the application of nanotechnology and nanoparticles (NPs) to medicine and cancer has revolutionized techniques for diagnosis and treatment, now offering a broad range of alternatives. In terms of diagnosis, magnetic nanoparticles (MNPs) are contemplated as MRI contrast agents [16,17] or organic and inorganic NPs as nano-biosensors [18,19]. In addition, NPs also play an important role in cancer treatment, improving chemotherapy delivery [20,21,22], and they are being used in the development of innovative techniques such as gene [23,24] and HT therapies. Another interesting approach is the use of NP-loaded cells instead of individual NPs, a good example being the use of MNP-loaded immune cells for magnetic targeting in adoptive cell transfer therapies [25]. Both the possibilities for diagnosis and treatment make these nanomaterials, and specifically MNPs, very powerful candidates in the fight against cancer, know referred to as theranostic agents [16,26]. The success of NPs can be largely explained by the possibility of using them as more specific guided medicines and from the growing number of clinical trials, the use of NPs in cancer is becoming a reality [27,28,29].

First of all, NPs have the intrinsic ability to accumulate passively in tumours due to an Enhanced Permeability and Retention (EPR) effect, a concept coined in 1986 [30]. This EPR effect is based on the disruption and consequently, the loss of impermeability in the tumour vasculature, allowing the extravasation of proteins and macromolecules, and also of NPs into the interstitial space of tumours [31]. Moreover, the absence of functional lymphatic vessels contributes to the retention and non-clearance of the NPs [32]. The use of NPs (or macromolecular drugs, polymeric drugs or liposomes) is crucial to obtain an adequate EPR effect, since the use of these nanocarriers allows the desired drug to reach and accumulate in the tumour avoiding renal clearance caused by its small size (renal clearance threshold = 40 kDa) [30]. The success of the EPR effect is not exclusively dependent on the size of the nanoparticle or nanomedicine, but is much more conditioned by the tumour environment: size and concentrations of endothelial cells fenestrations, grade of fibrinolysis or thrombocytopenia in tumour hypoxic area, pericyte coverage of tumour microvessels, amount of collagen IV in basement membrane or density of extracellular matrix, and it, in turn, it is closely dependent of the type of tumour and organism. Nevertheless, an adequate diffusion extravasation and retention of NPs with sizes larger than 100 nm would be compromised [32]. Another way to direct NPs to cancer cells is through their active accumulation. This strategy involves the functionalization of the NP surface with antibodies or other specific ligands that recognize and promote NP uptake into target cells [33,34]. Even local NP injection into the tumour is a less aggressive and more specific minimally invasive option than conventional therapies not based on NPs [32].

Returning to HT, the possibility of a rationally targeting NPs to cancer cells offers two significant improvements over non-specific therapies. On the one hand, treatment effectiveness is notably enhanced due to the fact that each loaded NP can act as an individual heat source, only increasing the temperature in the areas where NPs accumulate and not affecting the surrounding tissues. Consequently, the HT produced with NPs is associated with fewer side-effects. Moreover, research into NPs is still constantly refining their synthesis and preparation, which is becoming cleaner, faster and cheaper, as witnessed by their single-step synthesis by microwaves [35,36].

**Figure 1:**
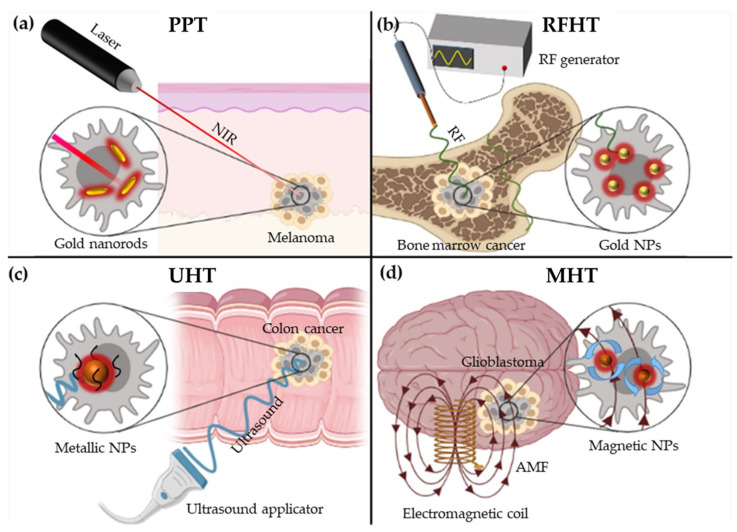
Therapies based on the accumulation of nanoparticles in the tumour and on the application of an external stimulus to induce nanoparticle heating: (**a**) Photothermal nano therapy (PTT), (**b**) Radiofrequency nano-hyperthermia (RFHT), (**c**) Ultrasound nano-hyperthermia (UHT) and (**d**) Magnetic nano-hyperthermia therapy (MHT).

Consequently, nanomedicine is particularly relevant to the future of HT therapies, which are generally based on the accumulation of NPs at target sites and the application of an external stimulus that induces NP heating. There are four different ways to achieve nanomaterial-induced MH, being the nature of the stimulus and the type of nanomaterials used (with their intrinsic properties) crucial parameters to achieve hyperthermia phenomena (Figure 1; Table 1).

#### 1.3.1. Photothermal Nano Therapy (PTT)

Photothermal nano therapy, usually shortened as photothermal therapy (PTT), is based on the capacity of agents to convert optical energy applied through a laser into heat. The laser beam can be tightly controlled, adjusting parameters such as the power density, duration or wavelength. Near-infrared (NIR) wavelengths (700–1400 nm) are normally used due to their good tissue penetration without producing undesirable damage or burns. Different nanomaterials can be used as photothermal agents but possibly, the best studied are gold NPs. When gold NPs are illuminated with NIR light, the energy produced by the excitation of their surface plasmon resonance (SPR) is transformed into heat and released to the local environment. The SPR effect is due to the oscillation of the free surface conduction electrons when excited by light, and they oscillate more with a resonance wavelength determined by the geometrical features of the gold nanomaterial [37,38]. For this reason, the size, surface and shape of gold-nanorods, and hence, their anisometry means they produce the best response to NIR excitation [39]. The PPT is a phenomenon shared by other nanomaterials, such as carbon nanomaterials (nanotubes, fullerene, graphene) [40,41] and CuS NPs [42] related to optical absorption. The effectiveness of PTT therapy is largely dependent on the penetration capacity of NIR, and it is more effective in melanoma and non-deep tumours. However, NIR-I (1000–1150 nm) penetrates well into tissues and organs like kidney, spleen and liver, whereas NIR-II (700–1000 nm) is the best option for muscles, and for stomach, heart and brain tissue in rats [43].

#### 1.3.2. Radiofrequency Nano-Hyperthermia Therapy (RFHT)

Radiofrequency nano-hyperthermia therapy (RFHT) is based on the capacity of different nanomaterials to absorb non-invasive radiofrequency electric fields (RF-EFs) and release heat in response [44]. The physical mechanisms explaining RFHT remain controversial and although Joule heating is considered the main mechanism, others will also contribute [45]. The longitudinal acoustic vibrational mode is another theoretical mechanism proposed to explain RFHT, for example involving the absorption of RF-EF energy by Fermi electrons [46]. Different nanomaterials release heat after exposure to RF-EFs, such as Pt or Si NPs and carbon nanotubes. However, the best studied are again Au NPs, for which high concentrations and small sizes have been demonstrated to be important parameters to enhance RFHT. The main advantage of this strategy is the greater penetration of RF-EF, which enables deep tumours to be treated. Indeed, RF therapies have been tested against bone marrow, liver, pancreas, colon or lung cancer [44].

#### 1.3.3. Ultrasound Nano-Hyperthermia Therapy (UHT)

Ultrasound nano-hyperthermia therapies (UHT) are based on the capacity of some nanomaterials to enhance the effects produced by exposure to ultrasound. Ultrasound therapy produces a non-specific response, such that extensive ultrasound exposure could provoke thermal damage to healthy tissues. However, high intensity focused ultrasound (HIFU, 0.1–1 kW/cm^2^) is a less harmful alternative [47]. The selection of nanomaterials used in UHT is crucial due to their importance to enhance the effects of ultrasound therapy where NPs are located. Ultrasound affects tissues in two ways: thermally and mechanically. In UHT the thermal interactions depend on the attenuation coefficient and thermal conductivity of the NPs. The first of these contributes to the absorption and scattering of the ultrasound waves so that large NPs provoke a major attenuation and therefore, greater thermal dissipation. In terms of conductivity, small metallic NPs are the best candidates to improve the thermal conductivity when cell are loaded with these NPs. On the other hand, NPs maximize the mechanical interactions, consequently the cavitation nucleation threshold decrease and cavitation phenomenon is induced, which causes mechanical cell-membrane damage and cell lysis. Theoretically, this therapy could be applied to all types of tumours without any restriction in terms of depth. However, in practice it has been little studied due to the lower specificity and the expensive equipment required, and it has mainly been carried out with silica, gold and iron-oxide NPs against breast, melanoma or colon tumours [44].

#### 1.3.4. Magnetic Nano-Hyperthermia Therapy (MHT)

Magnetic nano hyperthermia therapy (MHT) is essentially based on the intrinsic ability of MNPs to respond to alternating magnetic fields (AMFs) by converting magnetic energy into heat [37,44]. This approach is a valuable alternative for some kind of cancers located close to vital organs and in particular, for those difficult to remove surgically like some brain tumours [48]. This therapeutic approach allows the main clinical limitation of conventional radiotherapy to be overcome, that is the lack of selectivity of ionizing radiation which damages healthy and tumour tissues alike [49], thereby limiting the treatment to certain tumours. In MHT, the tumour cells (*in vitro*)/tumour (*in vivo*) are first treated with functionalized MNPs that are specifically internalized by these cells and then, the cells/tumour are subjected to an AMF to increase the local temperature of the tumour cells and induce controlled apoptosis. The main disadvantage for this therapy is having pre-clinical implementation problems and it is necessary a well-understanding actuation mechanism in order to exploit its full potential. Concretely, these handicaps are discussed in the point 5 of this manuscript: nanoparticles aggregation and consequently loss of ability to releasing heat.

The purpose of this review has not been to carry out an exhaustive search of all the articles published in recent years in medical databases that include the term “magnetic hyperthermia therapy”, but we have preferred to include those articles that we believe have contributed the most to understanding MNPs behavior in response to AMF in biological milieus, which is a critical step to drive magnetic hyperthermia therapy toward clinical implementation. The focus of this review is placed on the specific issues related to the physical basis of this therapy, the type of MNPs used and the tumours that are best suited to this therapy. The concepts underlying MHT will be addressed to understand what type of NPs are the best candidates to use. The biological effects of MHT at the cellular and molecular level will be explained and ultimately, the most promising and novel rationally improved strategies that currently produce the best results will be described. Finally, the current ongoing clinical trials will be reviewed and prospects for the clinical implementation of MHT therapies based on MNPs will be reviewed.

## 2. Physical Concepts of Magnetic Hyperthermia (MH)

**Figure 2:**
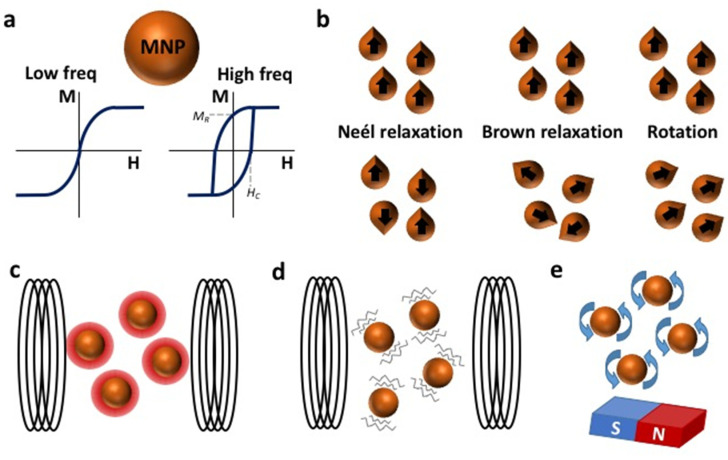
Physical properties of magnetic nanoparticles (MNPs), and the magnetic response to low and high frequency magnetic fields. (**a**) At low frequencies the MNPs present a S-like magnetic response with a high susceptibility (superparamagnetic) and they transit towards a hysteretic magnetic response when exposed to high frequency AMFs. (**b**) The MNPs may lose spontaneously their magnetization by Néel and Brownian relaxation when the applied field is subtracted or rotate coherently in response to a rotatory magnetic field. Through these mechanisms the MNPs can convert the energy of magnetic fields into (**c**) heat, (**d**) mechanical vibration or (**e**) local torque in the cell environment depending on the features of the field applied.

The clinical application of magnetic hyperthermia is referred to MHT. This therapy is based on certain physical principles that we consider important to explain below. MNPs present unique magnetic properties that can be taken advantage of to achieve selective contactless heating mediated by AMF. The ferromagnetic materials used in the production of magnets are characterized by the remanent magnetization (M_R_) they present even in the absence of a magnetic field. This magnetization is a consequence of the alignment of atomic moments in a specific direction defined by the anisotropy of the material (easy magnetization axis). When ferromagnetic materials are reduced to the nanoscale, thermal fluctuations become more and more important until the thermal energy surpasses the anisotropy energy of the NPs, making the magnetic moment flip between two bistable positions of the easy axis and leading to what is called superparamagnetic behaviour. As a consequence of these spontaneous fluctuations, the M_R_ of the MNPs disappears and the magnetization (M) presents a S-like reversible response to low frequency magnetic fields (H) like that indicated in Figure 2a. The time taken for the magnetic material to lose its magnetization is called the relaxation time [50].

MNPs recover a ferromagnetic response when the oscillation of the applied field is faster than the relaxation time of the magnetic moments (high frequencies) and the magnetization processes take place through dissipative loops. This dual response is of interest for their biomedical application as they become contactless nanoheaters. Thanks to the lack of the M_R_ of superparamagnetic MNPs in the low frequency regime, they can be prepared as a colloidal suspension, avoiding aggregation. Consequently, they can be injected intravenously without any fear of them obstructing capillaries [51,52]. In addition, once they are situated in the target tissue they can be remotely activated as nanoheaters by applying a high frequency field, producing minimal effects in the surrounding biological tissues where only a weak magnetic response is produced. In contrast to other contactless mechanisms of activation, such as that required in photothermal or photodynamic therapies, AMFs can penetrate the body with minimal attenuation, ensuring homogeneous field conditions in the whole tumour without any shadow effect [53].

There are two dissipative mechanisms by which MNPs may lose their magnetization when the magnetic field is removed: Néel relaxation and Brownian relaxation (Figure 2b). The former relaxation is associated with the inversion of the magnetic moments between the two directions of the easy axis magnetization, and it depends on the magnetic features of the MNPs. The latter is produced by the physical rotation of the MNP within the liquid media. In both cases, relaxation leads to the misalignment of the easy magnetization axes and consequently, a cancelation the global magnetization of the system. Brownian rotation is a consequence of the random interaction with the surrounding media and therefore, it is controlled by the hydrodynamic size, the temperature and the viscosity of the medium [54]. In both cases, relaxation leads to the misalignment of the easy magnetization axes and consequently, a cancelation the global magnetization of the system. MNPs will adopt a faster relaxation mechanism depending on their intrinsic properties and those of the surrounding medium [55], although combinations of both mechanisms may exist. It has been postulated that in certain AMF conditions MNPs may physically align their easy magnetization axis with the applied field before undergoing Néel relaxation [56].

It has generally been claimed that for translational MHT, MNPs design must focus on optimizing the mechanisms of Néel relaxation [57] since the natural MNPs-aggregation induced by contact with biological milieus (for instance, during lysosome encapsulation) and the high viscosity of these media blocks the Brownian effect [58]. However, the heat produced by MNPs with pure Néel relaxation requires tumour cells to have a high concentration of Fe in order to increase the temperature, which is difficult to achieve by intravenous injection and that has been resolved by intratumour injection [59].

An additional response of MNPs to an AMF is mechanical damage due to the magnetic torque generated in the presence of the field, which is in turn due to the misalignment between the field applied and the easy magnetization axis. This is a different concept to the Brownian relaxation indicated above. In this case, the magnetic moments of the different MNPs maintain their relative alignment but they are rotated collectively to reduce the angle between their magnetic moment and the field applied. Equation (1) shows how the magnetic torque (*T*) applied by a magnetic field (*B*) to a magnetic moment (*µ*) grows with the misalignment (*θ*) between them [60].

(1) T=μ·B·sin(θ)−6ηVHdθdt

This equation also takes into account the resistance to rotation produced by the medium depending on its viscosity coefficient (*η*), the hydrodynamic volume of the MNP (VH) and the angular speed of rotation (*dθ*/*dt*). The order of magnitude of the mechanical torque in normal conditions for a single MNP is 10^−21^ Nm [61]. However, the torque generated by the magnetic field is enhanced for an aggregate of MNPs [62], like those observed inside the cell’s lysosomes.

The most relevant parameters (*η*, *V_H_*, AMF and frequency, etc.) have been revised in detail to determine the transition between heat dissipative mechanisms and magneto-mechanical actuation. As a result, in viscous media the mechanical rotation induced by AMFs is mainly relevant for large MNPs (15–50 nm) and low frequency AMFs (<10 kHz) [63,64], although it can be exploited to exert effective mechanical torque on biological components [65]. This transition with AMF frequency becomes even clearer in the case of large anisometric NPs [63] and some empirical studies reported that AMFs induce a torque [66] or a physical movement of the MNPs [67] that may produce mechanical damage of the lysosome and cell membrane [68,69].

The use of rotatory fields is more convenient when inducing mechanical damage to cells. These fields maintain the MNPs magnetized throughout rotation and it is technologically simpler to generate homogenous fields of high intensity (>0.1 T) using permanent magnets. According to theoretical simulations, the torque induced by rotatory fields is 30-fold that created by AMFs [61]. Hence, many of the studies that seek to use MNPs to induce mechanical damage now focus on this kind of magnetic stimulus [66,70].

### 2.1. Determining the Heating Power of MNPs

One of the main limitations for the critical analysis of MHT measurements is how to determine the heating power of MNPs [71]. The Specific Absorption Rate (SAR) is an empirical parameter frequently used by radiological protection departments to regulate the amount of radiation absorbed by patients when exposed to radiofrequency fields and it is normalized to the mass of biological tissue irradiated in terms of W/g [72]. This parameter was taken by the MHT community to quantify the amount of magnetic energy transformed into heat by a suspension of MNPs. However, it is important to note that in the latter case, the temperature increase (ΔT/Δt) is generally normalized to the Fe concentration in the magnetic colloid (m_Fe_) according to Equation (2), where C_V_ is the specific heat of the colloid [73,74].

(2) $$SAR\left(W/g_{Fe}\right)=\frac{C_{V}}{m_{Fe}}\frac{\Delta T}{\Delta t}$$

It is also important to note that this is a system-dependent parameter that varies with the field intensity (H) and frequency (f) of the AMF. With the aim of standardizing such parameters and comparing the heating power of MNPs studied under different AMF conditions, an alternative parameter has been introduced, the Intrinsic Loss Power (ILP). This parameter divides the SAR by the frequency of the field and the square of the field intensity (Equation (3)).

(3) $$ILP\left(W/g_{Fe}\right)=\frac{SAR}{f\;H^{2}}$$

This definition is based on the theoretical model proposed that considers a linear response of the magnetic moment to the AMF applied [75]. This model predicts a linear dependence of the SAR on the frequency and a quadratic dependence on H, although this is only valid for highly anisotropic MNPs and a small H, and thus, it cannot be assumed as a universal parameter. Besides, recent double-blind experiments showed that the specific features of the experimental set-ups and the thermal curves analysed may produce inconsistent SAR values between laboratories [71]. A promising solution to achieve a global and consistent parameter to determine heating power is to measure the high frequency magnetic loop of the MNPs [76]. As indicated previously, the amount of heat dissipated by a collection of MNPs is strictly related to the area of the high frequency hysteresis loops (A), see Figure 2a right). Therefore, the theoretical SAR can be derived from the product of this area to the number of magnetic cycles per second, i.e., the frequency of the AMF (Equation (4)).

(4) SAR=A· f

Hysteresis loops are intrinsic to magnetic systems and they provide information about the magnetic response to AMFs of different frequencies and intensities. Therefore, they provide information about the dissipative properties of the MNPs that are independent of the thermal diffusion of the medium and do not depend on the thermal loses of the calorimetric system. However, the AC magnetometers required to study these are still scare and generally homemade [77].

The ideal field and frequency AMF conditions for MHT are also still to be defined. The former limit was established for the maximum field-frequency product as H × f ≤ 4.85 × 10^8^ A m^−1^s^−1^ based on the feeling of discomfort in irradiated subjects [78], a subjective test of patient comfort. New AMF application systems can concentrate the radiation in a restricted volume, reducing the radiation dose received by the patient and enabling more flexible limits to be proposed, ranging between 1.8 × 10^9^ A m^−1^s^−1^ [79] to 8.3 × 10^9^ A m^−1^s^−1^ [80], and up to 18.7 × 10^9^ A m^−1^s^−1^ [81].

### 2.2. Other Advantages of Using MNPs

An additional functionality of MNPs that enhances their applicability in MHT is the possibility to concentrate them in a certain region using magnets. When MNPs are magnetized with a magnetic gradient they minimize their energy by shifting towards the region of the maximum field. This principle has been exploited to concentrate magnetic nanoagents to a target superficial tumour by locating a set of permanent magnets in the proximal skin area. Using the same principle, MNPs have been used to label circulating tumour cells so that they can be concentrated and detected for an early diagnosis of metastasis risk [82,83]. More recently, the magnetic guiding of immune cells loaded with MNPs has been proposed as an advanced solution to reduced vascular accessibility [84], and as a means to activate mechanosensitive membrane receptors that inhibit cancer proliferation [85].

MNPs also present interesting properties for clinical imaging techniques, such as MRI. The strong permanent field used to align the magnetic moment of water protons magnetizes MNPs, creating local regions of enhanced magnetic fields in the tissues where these MNPs lie. The local field created by MNPs modifies the relaxation time of the surrounding water molecules [86]. This changes the MRI contrast of the tissue loaded with MNPs and offers an interesting pathway for personalized therapy. In addition, Magnetic Particle Imaging (MPI) has emerged as a promising technique to solve the incompatibility of a simultaneous MHT and MRI (mentioned in 1.2.4) and ideally achieve theragnostic NPs which are useful as heat generators for MHT, and at the same time, for diagnosis through real-time in vivo image during the therapy. MPI is an emergent image modality which works by detecting the nonlinear magnetization of the flipping MNP. MPI present several advantages: ideal penetration and signal-noise ratio, no view limitations, highly sensitive, linear and quantitative signal, high contrast, zero ionizing radiation, and safer and persistent. All this makes this technique an excellent non-invasive 3D tomographic imaging method to be combined with MHT for a real-time therapy image guided [15,87].

## 3. MNPs for MHT

Maximizing the heating power of MNPs is an interesting approach to minimize the dose required for effective HT therapy. In this section we will summarize the most important strategies to maximize the amount of heat dissipated by MNPs. Considering equation 4, it is easy to identify that the SAR can be maximized by increasing the frequency of the AMF, the area (A) of the high frequency hysteresis loops, or both parameters at the same time. The simplest strategy to increase A is to increase the magnetization of the sample. Taking magnetite (Fe_3_O_4_) as a reference for biocompatible magnetic materials, saturation magnetization can be increased by doping its crystalline structure with other transition metals. The magneto-crystalline structure of magnetite is an inverse spinel made of two sub-lattices of magnetic moments aligned in opposite directions that occupy octahedral and tetrahedral positions [88]. Due to the higher number of octahedral positions and the Fe^2+^/Fe^3+^ occupancies, the magnetic moment of these two sub-lattices is not compensated, which classifies magnetite as a ferrimagnetic material. Thus, in the presence of a magnetic field the octahedral and tetrahedral moments lie in parallel and antiparallel, respectively. As such, the global magnetization of magnetite can be enhanced by substituting Fe ions with other transition metals with a higher atomic moment, like Mn that occupies octahedral positions [89,90], or with transition metals like Zn that have no atomic moment and occupy tetrahedral positions. Both effects can be combined in ternary Zn_x_Mn_(1−x)_Fe_2_O_3_ ferrites to maximize the saturation magnetization [91]. However, it must be borne in mind that the magnetic coupling between the lattices might be affected when high concentrations of dopants are used to reduce the magnetic order in the MNP, averting the enhanced magnetization. Besides, such complex formulations compromise the homogeneity of the stoichiometry in the sample.

An alternative strategy involves widening the magnetic cycle by increasing the coercivity field (H_C_) of the MNP. The Hc is related to the field required to cancel the remanent magnetization and thus, to the anisotropy of the magnetic system. MNPs with a high magnetic anisotropy (*K*) are harder to magnetize but they dissipate more energy as their magnetization is reversed. If the AMF is not sufficiently intense, most of the magnetic moments of the system remain fixed in their easy magnetization axis without dissipating any thermal energy. Thus, it is necessary to reach an AMF threshold to partially or completely overcome the anisotropy barrier of the MNP. The anisotropy field (*H_K_*) is an interesting parameter to define such a threshold in theoretical simulations (Equation (5)).

(5) $$H_{K}=2K/M_{S}$$

According to the numerical simulations based on the Stoner-Wolfarth theory, MNPs requires an AMF higher than approximately 0.4 *H_K_* to begin heat dissipation, which reaches it maximum value when the AMF approximates to the *H_K_* [92]. At higher AMFs, magnetic moments reach saturation and heat dissipation does not further increases. This theory also establishes a direct relationship between the H_K_ and H_C_ (H_K_ = 0.48 H_C_) for a set of MNPs with randomly oriented easy magnetization axes, making this parameter an interesting link between theoretical simulations and empirical data [93]. In summary, increasing the magnetic anisotropy of the system can help increase the SAR as long as the intensity of the AMF applied is similar to the H_K_ of the system.

The magnetic anisotropy constant (*K*) is a composite of two components: the magnetocrystaline anisotropy and the shape anisotropy. The former is associated with the crystalline structure of the MNPs and the coupling between their atomic magnetic moments. The magnetocrystalline anisotropy of magnetite can be enhanced by doping the structure with other transition metals, such as Co [94]. These cobalt ferrite MNPs have a K value that is 2 orders of magnitude higher than magnetite [95], creating MNPs with magnetic moments strongly fixed in the easy magnetization axes. Heat dissipation for highly anisotropic MNPs like cobalt ferrites is generally produced by Brownian mechanisms, which might be not ideal for in vivo applications.

The second contribution to anisotropy is defined by the geometry of the MNP. Magnetic materials tend to minimize their magnetic poles by locating their magnetization along their longest axis, establishing the geometrical easy magnetization axis. The geometric anisotropy can be enhanced by creating anisometric nanostructures like nanocubes, nanorods and nanodiscs [63,96,97,98]. These nanostructures are of general interest as they are good mechano-transducers that may apply large torques on biological components although they must be prepared with reduced dimensions to preserve their superparamagnetic response [99,100]. An alternative to superparamagnetism is to create nanomaterials with an exotic magnetic order known as magnetic-vortex that can be mechanically manipulated and also presents a lack of remanence [69].

As a rule of thumb, increasing the saturation magnetisation (M_S_) of the sample is a good approach for AMF inducers that operate at low intensity and high frequency, whereas increasing the K of the system is more convenient for AMFs that operate in high-intensity and low frequency conditions [101]. Both strategies could be combined by creating magnetically coupled composites with two magnetic components [102]. The area of the high frequency hysteresis loops can be maximized by coupling a “soft” magnetic phase with the high M_S_ and a “hard” magnetic phase with large coercivity through an exchange interaction [103]. Indeed, CoFe_2_O_4_@MnFe_2_O_4_ core-shell MNPs represent a paradigmatic example of this kind of system [104]. The synergetic effect of such magnetic coupling generates an increase in the SAR value of one order of magnitude with respect to similar MNPs with a single ferrite phase (3.03 kW/g). Even more impressive results were obtained with Zn_0.4_Fe_2.6_O4@CoFe_2_O_4_ core-shell MNPs prepared with a cubic shape in which the SAR was above 10 kW/g [105]. Although these are outstanding SAR values compared to other MNPs [106], the synthesis of homogeneous core-shell structures with a controlled shell thickness remains a challenge.

A more feasible approach to exchange coupling to develop advanced magnetic nanoheaters is the preparation of multicore MNPs, also known as nanoflowers. These structures are made of aggregates of magnetic nanocrystals with common epitaxial interfaces that couple the magnetic responses of the nanocrystals. Exchange coupling creates a cooperative magnetization process between the nanocrystals formed that increases the susceptibility of the aggregated nanostructures [107]. This increase in susceptibility implies a rise in the SAR values up to c.a. 2 kW/g at very high frequencies (700 kHz), even at moderate AMF intensities (25 kA/m) [108]. The amount of heat dissipated strongly depends on the primary crystal size, and on the composition and extent of the interfacial surface [109], creating an energetic balance of the dipolar interactions and exchange coupling between the primary nanocrystals that may favour or hamper heat dissipation [110].

Dipolar interactions are also a crucial parameter when using MNPs as nanoheating agents. When MNPs are magnetized they create their own dipolar field that affects the magnetic response of the surrounding MNPs. This effect is especially relevant for highly concentrated colloids and in aggregated systems in which the MNPs are in close proximity, such as endosomes. For small MNPs, the dipolar field may increase the anisotropy barriers and enhance their heating performance [111]. But in most cases the effect is the opposite, the dipolar field cancelling the effect of the applied field, reducing the susceptibility of the system and consequently diminishing their SAR [112,113]. This has been postulated as a possible cause for the discrepancies observed in the heating performance of MNPs between ex vivo and in vivo studies [114]. Only in the case of elongated aggregates, such as chains, does the dipolar interaction result in cooperative magnetization that favours susceptibility and increases the coercivity of the system [115].

Although these parameters are interesting from the point of view of the magnetic properties, superparamagnetic iron oxide nanoparticles (SPIONs) are clearly the most advanced candidate in terms of commercial availability, regulation and clinical trials. In terms of magnetic properties, SPIONs present a relatively high susceptibility and low residual magnetization in the absence of an external magnetic field. Moreover, SPIONs are well-tolerated and they have low toxicity profiles, even in long-term studies [116]. In addition, they can be biotransformed from SPIONs to other iron compounds, facilitating their clearance [117,118,119,120]. SPIONs have been in clinical use for years, and several types have been approved for use in humans by the European Medicines Agency (EMA) and the Food and Drug Administration (FDA) in the USA, especially as anti-anaemic drugs and contrast agents for MRI [121].

### Preparation of Candidate MNPs for MHT

In designing MNPs for HT, a balance must be reached between the size of the magnetic core to maximize the heat released (>10 nm) and the colloidal stability in biological media required for intravenous injection (<50 nm). Above these sizes, magnetic interactions between NPs are very strong and it is difficult to keep them apart despite their coating, such that they tend to aggregate and precipitate. One NP formulation already approved for use in cancer therapy is NanoTherm^®^, approved in 2010 by the EMA to treat recurrent GBM and in 2018 by the FDA for human prostate cancer (https://www.magforce.com/en/home/about_magforce/#highlights; accessed on 27 July 2021). NanoTherm^®^ is a colloidal suspension of aminosilane coated 15 nm iron oxide NPs (with an iron concentration of 112 mg/mL) that can be delivered percutaneously into the tumour tissue. However, the challenge remains to develop NPs with enhanced specific loss of power and efficient delivery, within clinical AMF design constraints [122].

Iron oxide MNPs commercially available for HT are produced by precipitation of iron salts (Fe(II) and Fe(III)) in alkaline aqueous solutions. The size of the particles does not exceed 20 nm and it can be ensured by thermal treatment for long periods of time or by controlling the pressure in autoclaves. Thus, high pressure homogenisation processes allow the formation of individual crystals with mean diameters of 15–20 nm [123]. In a similar way, core–shell NPs and mixed ferrites are obtained by co-precipitation of stoichiometric mixtures of solutions containing divalent (Mn(II), Co(II), Zn(II)) and trivalent metals (Fe(III)) in alkaline medium [124]. Although core-shell MNPs can be finely tuned in organic media synthesis, the synthetic methods in aqueous media are preferred because they do not need additional coatings for water transference and are fully scalable to mass production, although controlling the size distribution and crystal order is limited due to the use of temperatures below 100 °C.

Controlling the shape of NPs and producing larger MNPs (>20 nm) can be achieved by thermal decomposition in organic media at temperatures as high as 300 °C, while cubes or rods are prepared using shape directing agents, either carboxylic acids or amines, respectively [96,97]. Autoclaves have recently been used to produce up to gram quantities of cubes, using iron pentacarbonyl as a precursor (Patent: WO2020222133A1). Other ferrites, such as manganese ferrite, zinc ferrite or a mixture of them in the form of core@shell or alloys prepared by this method, have showed excellent properties for HT [101].

Moreover, the assembly of magnetic cores into regular structures has been shown to significantly influence the HT behaviour of the particles, requiring the control of some key synthetic parameters that drive the self-assembly and growth process, such as surfactants and the viscosity of the medium. Thus, flower-like iron oxide assemblies between 25 and 250 nm can be obtained by heating a mixture of Fe(II) and Fe(III), or of an Fe(III) salt alone, to 200 °C in a heating mantel or in an autoclave [108,125]. In this sense, polyols are very interesting polar solvents that work as reducer, surfactant and high temperature synthesis media, allowing the use of inorganic salts as precursors [126]. Moreover, it is possible to combine the polyol procedure with more efficient heating technologies, such as microwave heating, leading to higher production yields over shorter reaction times [127].

The assembly of magnetic cores can be exploited even in the absence of an interface between them. The chains of magnetic nanocubes naturally produced by magnetotactic bacteria have for long been the best candidates for MHT, with a SAR of 2.38 kW/g at 310 kHz and 30 kA/m [128]. For this reason, several mimetic systems have been produced using silica as a template or other anisometric nanomaterials [129]. Finally, further coating and functionalisation is possible for NPs obtained in either aqueous, organic and polyol media. Coating with aminosilanes and/or polysaccharides is mainly used for targeted HT applications [130]. These coatings provide an excellent first layer for the bioconjugation of biomolecules, such as antibodies or peptides, drugs or biomarkers.

## 4. The Biological Effects of the Application of AFM to Cells Loaded with MNPs: Is It Always Hyperthermia?

Having established the physical principles and the materials used in the development of MHT, we can focus on the biological effects that they produce. However, rather than adopting a general or macroscopic view of this issue, we shall focus on the cellular and subcellular effects of this treatment. There are many in vitro and in vivo studies that have correlated MNPs and AMF therapies with extended life expectancy or tumour regression [131], although the biochemical mechanisms responsible for such improvements remain unclear. Depending on the magnetic features of the MNPs, and on the AMF amplitude and frequency, the MNPs can transform the energy of the magnetic field into heat or mechanical effects (Figure 2c, d, e). The application of the AMF may cause the magnetic moments of the internalized MNPs to rotate in the direction of the field (Néel’s relaxation) and the actual MNPs to physically rotate (Brown’s relaxation). These two responses to the magnetic field are manifested to a greater or lesser extent depending on the intrinsic characteristics of the NMPs (size, shape, anisotropy, crystallinity) and the magnitude of the AMF. When the AMF is applied and it alternates at high frequency, the continuous re-orientation of the MNPs with the magnetic field alters the release of heat by the MNPs [132] or the physical movement of the MNPs, provoking mechanical damage.

Consequently, there is a controversy around if it could be termed hyperthermia for all effects provoked by MNPs + AMF treatment. So that, we propose to classify the biological effects into four groups, depending on the main cause of the biological effects observed. First, we talk about the most intuitive mechanism, the rise of temperature, that is, hyperthermia. Then, we mention several studies where the authors believe that the resulting effects are not related to temperature, but due to the physical and mechanical MNPs movements. Later, we introduce a section to propose an explanation for these apparently non-temperature-related effects, offering the possibility of a macroscopically undetectable hyperthermia phenomenon. Finally, we mention other indirect treatment processes that help us to tumour regression.

### 4.1. Biological Effects of Heating

**Figure 3:**
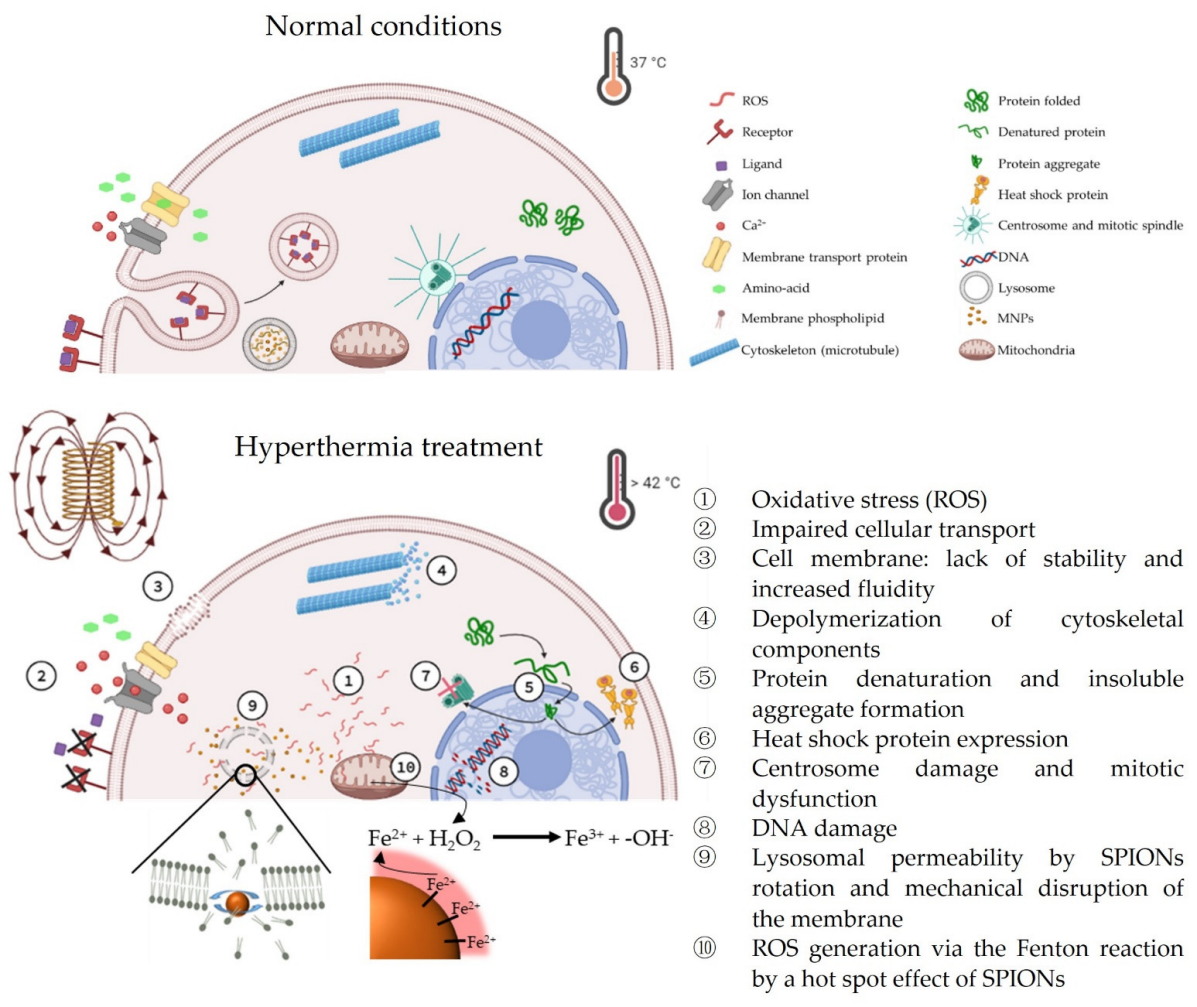
Cellular and sub-cellular biological effects derived from hyperthermia. **Upper:** normal conditions (37 °C). **Bottom:** hyperthermia treatment (>42 °C).

It is well known that a rise in temperature triggers cell death, yet not all cell types are equally sensitive. Cancer cells are considered to be more susceptible to HT than healthy cells due to their higher metabolic rates [133], the hyperthermic inhibition of DNA repair [134] and the poorer heat dissipation through the blood flow [135]. More specifically, the biological effects of HT include (Figure 3): an increase in oxidative stress (Figure 3. ①) [136,137]; inactivation of membrane receptors and increase in ion permeability that affects cell transport (inhibition of amino acid transport and increased Na^+^, K^+^, and especially Ca^2+^: Figure 3. ②); a lack of stability and an increase in membrane fluidity (Figure 3. ③); changes in cytoskeletal organization, involving microtubule, microfilament and intermediate filament depolymerization (Figure 3. ④); increased protein denaturation and insoluble protein aggregation in the nucleus (Figure 3. ⑤), which promotes heat shock protein expression (Figure 3. ⑥) and centrosome damage, as well as mitotic dysfunction (Figure 3. ⑦); and eventually, DNA damage or denaturation can occur (Figure 3. ⑧) [138].

### 4.2. Biological Effects of Mechanical Rotation or Vibration

It is interesting that there have recently been several reports of cell death after MHT without any perceptible rise in temperature [139,140,141,142]. The mechanisms responsible for these effects have not yet been elucidated, although there is data indicating that they are related to an increase in lysosomal permeability, which triggers an enhanced reactive oxygen species (ROS) production and enhanced activity of the lysosomal protease cathepsin D in the cytoplasm diminishing tumour cell viability [47,143,144]. This lysosomal permeabilization might be also caused by mechanical rotation or vibration of SPIONs altering lipid membrane stability (Figure 3.⑨). Indeed, dynamic magnetic fields induce a slow rotation of lysosome targeted SPIONs, tearing the lysosomal membrane and activating apoptosis [145]. Moreover, according to theoretical simulations, the rotation of MNPs in a liquid media can be induced by either rotatory fields or AMFs. However, at moderate field intensities the torque induced by rotatory fields is 30 times higher than those created by AMFs [61]. Evidence of the mechanical damage produced by MNPs under an AMF has come from reports of lysosomes rupture inside the cells [144].

### 4.3. Biological Effects Derived from Non-Perceptible Heating: The “Hot Spot” Effect

Another possible explanation for the biological effects of AMFs that are apparently unrelated to temperature changes might be very local intracellular heat release from SPIONs (not detected macroscopically), known as a “hot spot” effect. This local heat-release enhances biological effects, such as the generation of ROS by the iron oxide surface of the NPs through the Fenton reaction (Figure 3.⑩), which is known to be accelerated directly by temperature [146]. Hence, we think that markers of sub-cellular temperature rises (e.g., Hsp70 [147] or thermal nanoprobes) should be implemented routinely in these studies to determine if tumour cell death can be always attributed to HT (even if these occur on a subcellular scale), or whether the biological effects that occur are unrelated to temperature.

### 4.4. Biological Effects Derived from Other Indirect Process

It seems clear that independently of whether they are due to MNP heating or their mechanical rotation and vibration, biological effects not directly or not exclusively related to temperature play a crucial role in tumour regression. ROS formation through Fenton reactions is probably the best studied of these, given that ROS can severely damage cell elements due to oxidation, such as DNA, proteins, lipids and enzyme cofactors, thereby inducing apoptosis [148]. ROS formation can occur by lysosome degradation or through the breakdown of other subcellular structures but also, by MNP interactions and Fenton reactions at the MNP’s surface. Interestingly, and concomitant with ROS production, an increase in fluidity and a loss of cytoplasmic membrane integrity also activates cell death, either necrosis or apoptosis [5]. Other effects not directly related with the MNP-tumour cell interaction have an enormous importance in the fight against cancer. MHT has also been associated with activation of DCs and NK cells [149].

## 5. MNP Behaviour in Response to AMF in the Biological Milieu

In the previous section, we have analyzed the biological effects driven directly or indirectly by MNP exposure to AMF in MHT settings, that may or may not be trigger by MNPs heat release in response to AMF exposure. In recent years, many studies have suggested that the ability of MNPs to produce heat in response to AMF exposure when MNPs are in biological milieus is severely reduced, even sometimes undetectable, a circumstance that would not be desirable for MHT therapies. A possible explanation for this undesired behaviour could be that the magnetic response of MNPs to AMF was modified as a consequence of MNPs-cell interaction [150,151], being the causes of these changes in the magnetic properties a reduction in MNPs mobility, dipolar interactions, milieu viscosity, and MNPs clustering or aggregation [58,114,150,151,152,153]. This alteration of MNPs magnetic properties implies a dramatic reduction of SAR values, that could be observed when MNPs are aggregated by contact with cells, but also by contact with physiological milieus or viscous media emulating cellular environment, and depending on the intrinsic properties of the MNPs, these SAR decreasing values could be more than 60% [114,152], even in MNPs that, after being tested in aqueous medium, showed a promising heating capacity [153]. Most of the observed alterations in the MNPs magnetic properties could be explained by the restriction of the Brownian relaxation, since MNPs cannot respond to AMF with rotation because MNPs are physically immobilized and blocked. Therefore, when Brownian relaxation component is suppressed in biological conditions, and it was demonstrated that Néel relaxation was unaffected by changes to their biological microenvironment, emphasizing the importance of MNP intrinsic magnetic properties for MHT when particle mobility cannot be kept. So that, Néel relaxation component becomes the only possible heat induction mechanism [58]. Likewise, doping MNPs with Zn allows a strong Néel relaxation that was preserved after MNPs-cell interaction, which is suitable for heat releasing in MHT [151].

Although Néel relaxation contributes for heating generation during MHT the most important component for heating during an ideal MHT is the Brownian relaxation. Therefore, it is a key point to understand why the MNPs are immobilized in and if it is a reversible process. It has been shown that MNPs that were blocked as a consequence of cell internalization, can recover their original magnetic properties, including the Brownian relaxation, when the cells that contained them were lysed, due to the integrity of the magnetic core is preserved during this process [57] suggesting that MNPs immobilization or aggregation is the final cause of loss of Brownian relaxation. In another study, three systems with MNPs different spatial distributions and grades of aggregation were analyzed in order to compare their magnetic properties: isolated MNPs, MNPs-liposomes system, and MNPs-cell interaction (using, in turn, Jurkat cell line that attached MNPs to the outer membrane and Pan02 cell line that internalized the MNPs). Results showed that the biological environment played a crucial role in the dynamic magnetic response of the MNPs, being more altered for MNPs-cell system, and concluding that the simple fact of being in contact with the cells triggers MNPs aggregation [154].

Other studies tried to determine if this aggregation derived from MNPs-cell contact was a process dependent on the intrinsic properties of the MNPs or the host cell line. For that, co-precipitated maghemite nanoparticles, assembly of the same maghemite nanoparticles in liposomes, cobalt ferrite nanoparticles, iron oxide/gold dimers, iron oxide nanocubes and iron oxide nanoflowers were tested in three different biological environments: MNPs in water, MNPs attached to adenocarcinoma SKOV-3 cells membranes, or MNPs internalized in SKOV-3 cells. As result, a rapid fall in the heating capacity of all the nanomaterials tested (regardless of its different composition, shape or size) has been observed when MNPs were associated with the cell membrane or were internalized [153]. Likewise, different core size MNPs (6, 8 and 14 nm), coating (APS: 3-aminopropyltriethoxysilane, and DMSA: dimercaptosuccinic acid), cell line (Jurkat and Pan02) and subcellular localization (membrane or internalized in endosomes/lysosomes) were tested in biological milieus being demonstrated that the aggregation process was independent of MNPs core size, coating, cellular environment, host cell line and MNPs subcellular localization [150].

Currently, these problems of aggregation or blockade of MNPs are the main bottlenecks in MHT therapies, so that more efforts will be required to develop strategies directed to avoid them to achieve a satisfactory MHT.

## 6. Rational Design of Strategies Based on MNPs for MHT and Their Applications to Tumours *In Vitro* and *In Vivo*

**Scheme 1:**
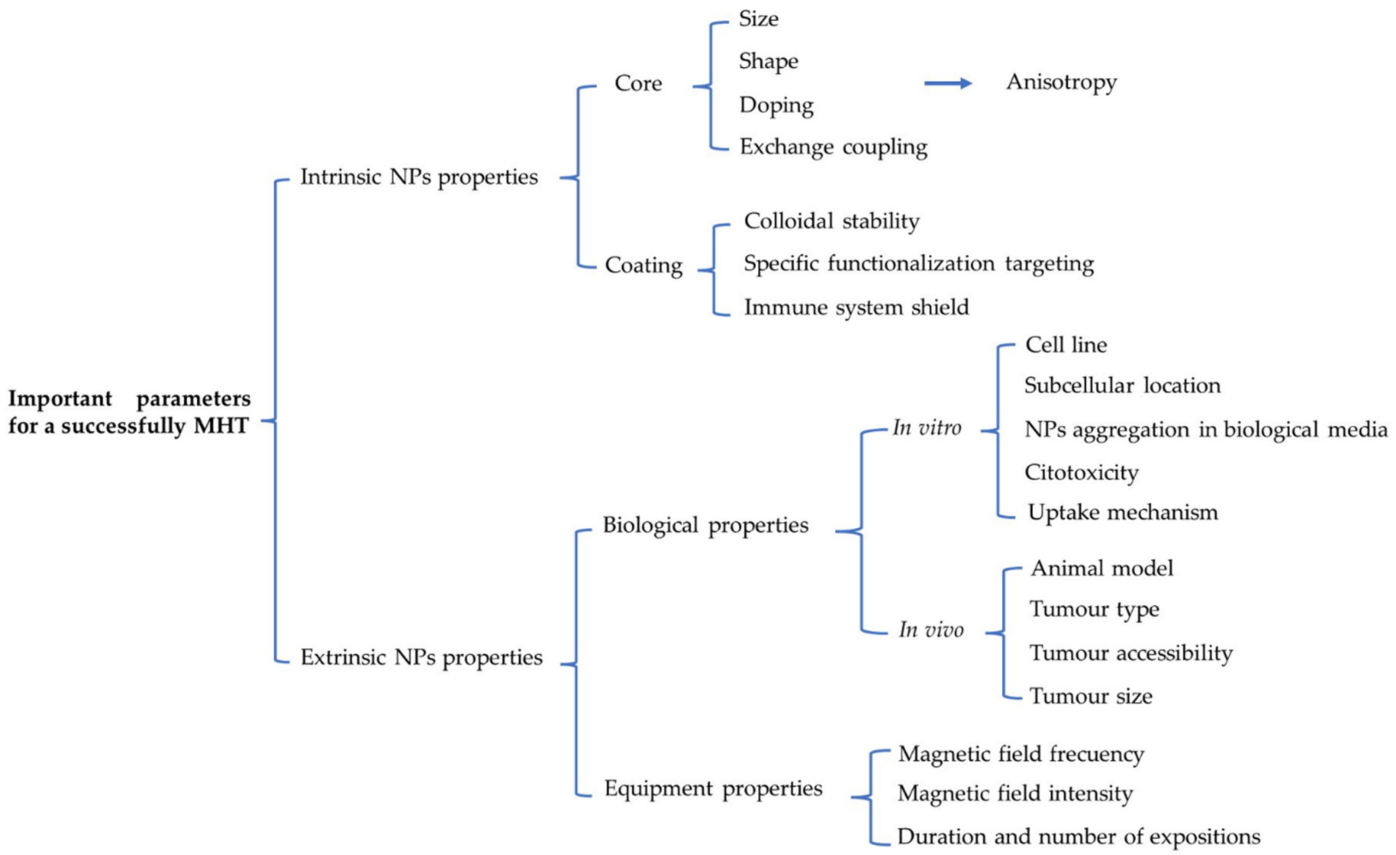
Main parameters to be considered to achieve a successfully magnetic hyperthermia treatment.

Having described the physical and biological behaviour of MNPs in relation to MHT, we shall assess how this knowledge has been directed towards extracting the full potential of this therapy through the rational design of MHT strategies based on MNPs. Hence, it is important to consider the different intrinsic (size [155], shape [156], doping, etc…) and extrinsic (magnetic field intensity and frequency, subcellular location, intracellular aggregation, etc…) parameters that govern the success of such treatments (Scheme 1). As such, the different studies where MHT has been successfully achieved based on the rational design of in vitro and in vivo experiments will be considered.

### 6.1. Fine-Tuning the Intrinsic and Extrinsic MNP Properties for In Vitro Magnetic Hyperthermia

We can find many examples of rationalized MNP designs that have been considered for in vitro MHT. For instance, size-optimized MNPs enhance cell death in Jurkat cells after MHT [157]. Likewise, phospholipid-PEG coating has been used to concurrently deliver Doxorubicin and to generate heat for an enhanced multimodal cancer treatment in HeLa cells [158]. Likewise, MNP functionalization with the folate-receptor (a tumour marker) has been employed for smart delivery to the MCF7 and G1 cell lines, with no uptake by a control L929 cell line [159]. Furthermore, it has been described that MNPs pegylation coating is capable of counteracting the interactions between dipolar particles while maintaining a low level of nanoparticle aggregation in environments of different ionic strengths and viscosities [160]. Each of these serves as a good example of the rationalization of MNP size, coating and targeting for in vitro MHT.

We carried out studies that combined different strategies for the rational design of MNPs [161], synthesizing them by thermal decomposition to obtain 18 nm flower-like Mn-doped SPIONs covered with DMSA and functionalized with cRGD (an αvβ3-Integrin-Ligand) peptide (from now on named NF-DMSA-PEP) that targets the U87MG glioblastoma cell line. These NF-DMSA-PEP had higher SAR values than 12 nm spherical MNPs covered with DMSA (NP-REF) and 20 nm flower-like MNPs covered with citric acid (NF-REF), demonstrating the notable role of rationalized intrinsic MNPs properties (size, shape, doping and coating MNPs). Furthermore, extrinsic properties related to the biological features of the target cells must also be considered. MNPs uptake was tested by comparing our NF-DMSA-PEP system with the same MNPs without the cRGD peptide (NF-DMSA). NF-DMSA-PEP uptake by U87MG cells was enhanced 5–6 fold, while endocytosis-exocytosis cycles avoided compact aggregation inside lysosomes and the resulting decrease in NP-induced HT. Consequently, peptide effectiveness was demonstrated, establishing 2 h as the optimal time to then apply the magnetic field. Finally, a 2 h NF-DMSA-PEP incubation followed by a 1 h AMF application (25 kA/m, 250 kHz) efficiently induced intracellular cell heating (Hsp70 over-expression), ROS production and cell death (but without inducing apoptosis). The biological effects observed were always stronger with NF-DMSA-PEP than with control MNPs.

### 6.2. Tuning Intrinsic and Extrinsic MNP Properties for In Vivo Magnetic Hyperthermia

One further step in the study of these approaches is the application of rational design to treat tumours in animal models, mainly glioblastoma, pancreas, breast and prostate. The most common way of administering nanoparticles for antitumor hyperthermia treatments is by intratumoral injection of MNPs, with or without the aid of the use of advanced imaging techniques to deliver MNPs into the tumour [162,163]. Systemic administration of MNP through intravenous injection followed by the biological or physical targeting of those MNPs to the tumour is another possibility, especially in the case of hard-to-reach tumors. However, MNPs doses needed is greater than for intratumoral administration, because the amount of MNPs that reach the tumour depend on several factors such as biodistribution [51], EPR effect [31], active targeting [33,34] and renal clearance [31]. These factors must to be taken account during the rational design process of the MNPs for MHT. For instance, the pegylation of MNPs allows long blood circulation times avoiding the rapid uptake by mononuclear phagocytic system and renal clearance [164]. Moreover, thinking in translational therapy, several studies conclude that EPR effect work properly in rodents but not in humans [32] making essential the use of active targeting strategies that could make the process more complex. Nevertheless, satisfactory MHT through rational designed MNPs intravenously administered has been done, as exemplified a study where a rational design based on pegylation of MNPs and functionalization with c(RGDyK) peptide solved the renal clearance and active targeting issues respectively, and finally glioblastoma regression in mice was achieved [165].

For intratumoral administration rational MNPs design is also needed to improve the regression of tumours. For instance, MNPs covered with DMSA and functionalized with doxorubicin as chemotherapeutic agents were administered intratumorally achieving breast cancer xenograft regression through a synergic effect [166]. Likewise, the development of a biocompatible magnetic lipid nanocomposite vehicle for encapsulate MNPs and doxorubicin was demonstrated to provoke a synergic effect sensitizing the tumour cells to cancer chemotherapy in a subcutaneous melanoma mice model [167]. Another example is the design of Janus MNPs charged with doxorubicin achieving the decrease of tumour weight in subcutaneous breast solid tumour models [168]. Another example, cubic-shaped MNPs, since cubic-shaped MNPs are better heaters than spherical MNPs [97], coated individually with a polymer shell to avoid MNPs aggregation, have been shown to be effective heat mediators for MH and heat-mediated chemotherapy on an in vivo xenograft tumour model using A431 epidermoid carcinoma cells [169].

In addition to the intrinsic properties of MNPs, the extrinsic properties must be considered. It was shown above that controlling and optimizing the biological parameters related to the MNP-cell interactions is important for satisfactory MHT. However, since intra-tumour injection is normally chosen in vivo, it is more interesting to consider other parameters like the animal model or the type, size and location of the tumour. In addition, optimizing the HT equipment conditions will play an even more crucial role. Modifying the magnetic field frequency and intensity will allow the MNP induced heating capacity be fine-tuned, with a higher frequency and a stronger magnetic field intensity translated into more heating, which is crucial for certain nanomaterials. Normally, MNPs require a threshold field to open their hysteresis loop and then achieve heat release. Nevertheless, higher magnetic field frequencies and intensities are not permitted for in vivo or translational therapies and thus, a compromise between these parameters must be found in order to remain within the safety limits [79,80,81]. The duration and the repetition of applications are other parameters to be considered. A full comparison of the in vitro and in vivo conditions for MHT has recently been prepared by Vilas-Boas et al. [170].

## 7. From the Laboratory to the Clinic

Despite the improvements implemented and the exponential growth of studies into MHT to treat cancer, translational investigation in this area has not progressed as desired and its clinical implementation has not yet occurred [79]. Various factors have influenced this delay, technological challenges being the most important. As mentioned previously, only one NP formulation has currently been approved for HT: NanoTherm^®^ (MagForce AG, Berlin, Germany). This sole alternative rules out the possibility employing a rational design of NPs with better physical characteristics and a potentially improved heating capacity at the same concentrations or doses [171]. In addition, NanoTherm^®^ is a ferrofluid that agglomerates in the tissue, and entrapment by macrophages as opposed to glioblastoma cells might result in cancer cells receiving insufficient doses [172]. Moreover, intratumour NPs injection must be employed to avoid aggregation, restricting their use to solid and accessible tumours like glioblastoma or prostate cancer. Consequently, a homogeneous NP distribution and therefore, constant heat distribution across the tumour is difficult to achieve [171]. Moreover, the inability to use active rational design also translates into a loss of effectiveness. On the other hand, the AMF applicator MFH 300F^®^ (later implemented as Nanoactivator^®^ F100: MagForce s AG, Berlin, Germany) is the only apparatus used in the clinical trials carried out to date, and always operating at a fixed frequency of 100 kHz and with a field strength of 0–18 kA/s [79]. However, changes to these parameters might produce better results while still respecting the safety limits.

**Figure 4:**
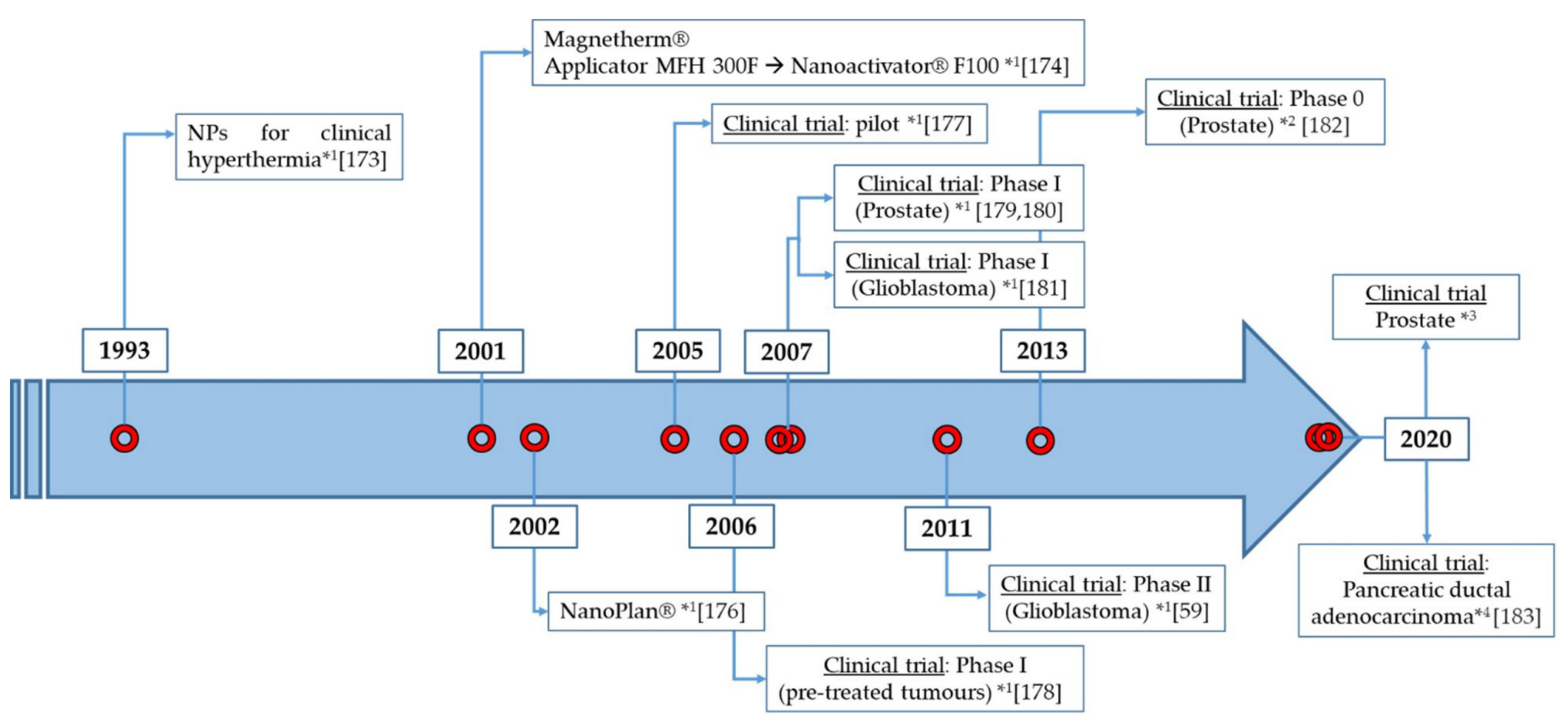
Timeline of the major milestones and clinical trials carried out on humans where Magnetic Hyperthermia Treatment was employed.

Several clinical trials have been perfromed by Charité—Universitätsmedizin Berlin (Germany) and the spin-off company MagForce AG, Berlin (Germany). The pipeline that has driven the initial idea towards the realization of clinical trials can been easily traced (Figure 4). In 1993, the potential use of SPIONs for HT therapy was first noted and how NP application localised to a tumour might be less invasive than other techniques. With clinical implementation in mind, a moderate concentration of ferrite 5 mg/g tumour was considered, coupled to clinically acceptable magnetic fields that were comparable to radiofrequency heating by local application and superior to regional RF heating [173]. Nearly a decade later, in 2001 this idea was developed further and a new magnetic field therapy system was introduced for the treatment of human solid tumours with magnetic fluid HT. In this study, two of the three pillars of HT therapy used in the Charité-MagForce clinical trials were well defined. Firstly, the aminosilane magnetite NPs used were subsequently manufactured by MagForce AG and called NanoTherm^®^ and they were seen to be more significantly taken-up by malignant cells than normal cells. Secondly, the first prototype of a magnetic fluid hyperthermia (MFH) therapy system was designed (Applicator MFH 300F) and which was later developed as Nanoactivator^®^ F100 by MagForce AG [174,175]. Finally, and just one year later the third pillar appeared, the software initially called HyperPlan and now NanoPlan^®^ (MagForce AG) that enables treatments to be planned through a thin-sliced CT or MRI scan. The software developed, in combination with the AMIRA^®^ visualization package (Mercury Computer Systems, Berlin, Germany), allows us to obtain 3D reconstructions of the NP distribution in the tumour and the localization of the thermometry catheter. Moreover, the physician can modify the parameters to simulate different scenarios and to determine the optimal magnetic field strength for the treatment, estimating the possible temperature distribution during the treatment [176].

Once the basic concepts have been fixed and a well-defined route obtained, a pilot clinical trial was carried out. The aim of this pilot study was to evaluate whether the MFH technique can be used for minimally invasive treatment of prostate cancer. The results indicated that HT using MNPs injected transperineally into the prostate was feasible and well-tolerated. Moreover, NPs were retained for at least 6 weeks in the prostate, making sequential HT treatment possible without the need for new NP application. This study formed the basis on which future clinical trials could be designed [177].

In the following years different clinical trials were performed, the details of which are shown in Table 2. These clinical trials focused on understanding, optimizing and improving particular aspects of the technique to enhance the results. First, in 2006 a phase I trial was performed to evaluate the feasibility and tolerability of thermotherapy using MNPs in different pre-treated tumours, as well as testing three different NP injection methods. The results showed that magnetic fluid and thermotherapy treatment was well-tolerated, with no or only moderate side-effects, respectively. Moreover, there was a clear need to further improve the temperature distribution by refining the implantation techniques, or simply by increasing the amount of NPs or the magnetic field strength [178].

A year later, a phase I trial was carried out in 2007 to investigate the feasibility of using thermotherapy with biocompatible SPIONs in patients with locally recurrent prostate cancer, evaluating an image-based approach for the non-invasive calculation of the 3D temperature distribution. It was concluded that heating using MNPs was feasible. Hyperthermic to thermoablative temperatures were achieved in the prostate at 25% of the available magnetic field strength, indicating the potential to reach higher temperatures and that a specific non-invasive thermometry method could be developed that may be used for thermal dosimetry [179]. In the same clinical trial, the treatment-related morbidity and quality of life (QoL) during thermotherapy was studied, and it appeared that interstitial heating using MNPs was feasible and well-tolerated by patients with locally recurrent prostate cancer. Furthermore, deposition of NPs in the prostate was evident 1 year later, even though a homogeneous distribution was not achieved. Finally, a refinement of the technique was needed to enable higher magnetic field strengths to be applied [180].

In parallel, a phase I trial was presented in 2007 to evaluate the feasibility and tolerability of the newly developed thermotherapy, using MNPs to treat recurrent GBM and guiding the intratumour NP injection by 3D imaging. The study demonstrated that thermotherapy using MNPs is safe to use in the treatment of brain tumours and that therapeutic temperatures ranging from HT to thermoablation can be achieved. These promising results opened the door to further studies [181] and consequently, a few years later a phase II clinical trial was carried out on 59 GBM patients. The objective of the study was to determine the efficacy of intratumour thermotherapy in conjunction with fractionated stereotactic radiotherapy for glioblastoma. This clinical trial confirmed the aforementioned advantages, demonstrating MHT to be a safe and well-tolerated cancer therapy. Moreover, the clinical trial concluded that the combination of HT and radiotherapy was clinically effective, augmenting the overall survival of patients. Finally, it was proposed that the combination of HT and chemotherapy (particularly temozolomide), in conjunction with intratumour NP targeting using convection-enhanced delivery (CED) is a promising approach for the treatment of other solid tumours that should be evaluated in future clinical trials [59].

Finally, the “Magnetic Nanoparticle Thermoablation-Retention and Maintenance in the Prostate: A Phase 0 Study in Men (MAGNABLATE I)” was the first clinical trial performed outside of the Charité –MagForce collaboration, promoted by University College London Hospitals (NCT02033447 [182].

## 8. Conclusions

From this review we can draw some important conclusions regarding the clinical implementation of MHT. In the case of MNPs, there are many studies about how their intrinsic properties (size, shape, composition, coating, etc) can affect their ability to generate heat in response to AMFs in non-biological milieus. This knowledge can be used to improve their rational design for MHT therapies to obtain the better heat release. However, the behaviour of MNPs in response to AMF in biological media, and the underlying cellular mechanisms that are triggered, are not yet fully understood, representing a major bottleneck in the application of MHT under physiological conditions. Therefore, it is important that more studies focus on the behaviour of MNPs inside cells. For example, it is important to study MNP biodegradation in different cell types and tissues, since MNP degradation in cells could affect their magnetic properties during treatment, and issue that could be critical in therapies that require the repeated application of the magnetic pulses long after the administration of NPs. In terms of the development of HT equipment, it is important to emphasize the importance of selecting the appropriate field intensity and frequency for each case, so that treatments produce specific effects. For clinical applications, it should be remembered that safety limits exist above which we cannot operate.

In general terms, the clinical implementation of MHT has not progressed as might have been anticipated, despite the success of some clinical trials. The global conclusions that we can extract from the clinical trials carried out to date is that the first steps taken have yielded promising results. However, in recent years no more clinical trials have been carried out. Fortunately, this tendency seems to be changing and a few months ago, a new and very ambitious pivotal single-arm clinical study for the focal ablation of intermediate-risk prostate cancer using NanoTherm^®^ was presented by MagForce USA, Inc. following its FDA approval. Along similar lines, in Europe both the Vall d’Hebron University Hospital and the Fuenlabrada University Hospital are involved in a new feasibility study on treating locally advanced pancreatic ductal adenocarcinoma (PDAC) as part of the NoCanTher project [183].

In summary, we strongly believe that increasing knowledge in the key biological aspects above-mentioned is highly necessary to achieve fine control of the process that could trigger the desired clinical implementation of MNPs-based magnetic hyperthermia.

## Figures and Tables

**Table 1:** Main features of the different nanotherapies.

	Nanotherapy			
	PTT	RFHT	UHT	MHT
**Equipment**	Laser	Radiofrequency generator	Ultrasound applicator	Electromagnetic coil
**External stimulus**	NIR	RF-EF	Ultrasound	AMF
**Physical principles**	Surface Plasmon Resonance (SPR) or Optical absorption	Joule heating and Fermi electrons	Thermal interactions and mechanical interactions	Néel relaxation and Brown relaxation
**Underlying effects**	-	-	Cavitation	NPs Rotation
**NPs employed**	Mainly: Au nanorods Others: CuS NPs or carbon nanomaterials	Mainly: Au NPs Others: Pt, Si NPs or carbon nanotubes	Si, Au or iron-oxide NPs	MNPs (SPIONs)
**Type of cancer treated**	Non-deep tumours: Melanoma	Bone marrow, liver pancreas, colon or lung cancer	All types of cancer (breast, melanoma, colon, etc…)	Mainly glioblastoma and Pancreatic cancer
**Main advantage**	Feasible and cheap	Great penetration	No depth restriction	High specificity
**Main disadvantage**	Limited penentration	Invasive (needle insertion)	Lower specificity Expensive equipment	Lack of response in biological milieus (see point 5)

**Table 2:** Summary of the different MHT clinical trials carried out by Charité –MagForce.

Trial Phase	N° of Patients	Cancer Type	NPs Used and Route of Administration	MFA and Conditions	Hyperthermia Sessions, Duration and Tª Reached	Year / Reference
I (Pilot)	1	Prostate	NanoTherm^®^ injected transperineally into the prostate (NPs retained for at least 6 weeks).	Applicator MFH 300F: (f = 100 kHz) H = 4.0–5.0 kA/m	Hyperthermia, six 1-h sessions: - 1st session: 40.0–48.5 °C - 6th session: 39.4–42.5 °C	2005 [177]
I	22	Different pre-treated recurrences tumours: chondrosarcoma, rectal carcinoma, cervical carcinoma, prostate cancer, sarcoma, rhabdomyosarcoma, ovarian carcinoma	NanoTherm^®^ injected by 3 implantation methods: - Infiltration under CT fluoroscopy (group A) - TRUS (transrectal ultrasound)— implantation guided by X-fluoroscopy (group B) - Intra-operative infiltration under visual control (group C)	Applicator MFH 300F: (f = 100 kHz) - H in pelvis = 3.0–6.0 kA/m - H in thoracic and neck up to 7.5 kA/m - H in head > 10 kA/m	Hyperthermia (six 1-h sessions) + radiotherapy/chemotherapy: - Tª ≥ 40.0 °C: 86% of tumour volume - Tª ≥ 43.0 °C: 30% of tumour volume (Group A) and 0.2% (Group B)	2006 [178]
I	10	Prostate	NanoTherm^®^ injected transperineally into the prostate guided by TRUS/fluoroscopy (89.5% were retained for at least 6 weeks and NPs still observed 1 year later).	Applicator MFH 300F (f = 100 kHz) H = 2.5–18.0 kA/m	Hyperthermia, six 1-h sessions: - Median prostate Tª: 40.1 °C (7.8 min at 43 °C in 90% of cases) - Maximum prostate Tª: 55.0 °C - Median urethral Tª: 40.5 °C - Median rectal Tª: 39.8 °C	2007 [179,180]
I	14	Glioblastoma multiforme	Intratumoural injection guided by 3D image of 0.1–0.7 mL of NanoTherm^®^	Applicator MFH 300F (f = 100 kHz) H = 2.5–18.0 kA/m	Hyperthermia (six 1-h sessions) + radiotherapy/chemotherapy: - Intratumoural Tª: 42.4–49.5 °C	2007 [181]
II	59	Glioblastoma multiforme	Intratumoural injection guided by 3D image of 0.5–11.6 mL of NanoTherm^®^	Nanoactivator^®^ F100 (f = 100 kHz) H = 2.0–15.0 kA/m	Hyperthermia (six 1-h sessions) + radiotherapy/chemotherapy: - Median Tª = 51.2 °C	2011 [59]
